# Reliability, construct and discriminative validity of clinical testing in subjects with and without chronic neck pain

**DOI:** 10.1186/1471-2474-15-408

**Published:** 2014-12-04

**Authors:** René Jørgensen, Inge Ris, Deborah Falla, Birgit Juul-Kristensen

**Affiliations:** Department of Physiotherapy, University College South Denmark, Esbjerg Ø, Denmark; Research Unit for Musculoskeletal Function and Physiotherapy, Department of Sports Science and Clinical Biomechanics, University of Southern Denmark, Odense M, Denmark; Pain Clinic, Center for Anesthesiology, Emergency and Intensive Care Medicine, University Hospital Göttingen, Göttingen, Germany; Department of Neurorehabilitation Engineering, Bernstein Focus Neurotechnology Göttingen, Bernstein Center for Computational Neuroscience, University Medical Center Göttingen, Georg-August University, Göttingen Germany; Department of Health Sciences, Bergen University College, Institute of Occupational Therapy, Physiotherapy and Radiography, Bergen, Norway

**Keywords:** Neck pain, Reliability, Validity assessment

## Abstract

**Background:**

The reliability of clinical tests for the cervical spine has not been adequately evaluated. Six cervical clinical tests, which are low cost and easy to perform in clinical settings, were tested for intra- and inter-examiner reliability, and two performance tests were assessed for test-retest reliability in people with and without chronic neck pain. Moreover, construct and between-group discriminative validity of the tests were examined.

**Methods:**

Twenty-one participants with chronic neck pain and 21 asymptomatic participants were included. Intra- and inter-reliability were evaluated for the Cranio-Cervical Flexion Test (CCFT), Range of Movement (ROM), Joint Position Error (JPE), Gaze Stability (GS), Smooth Pursuit Neck Torsion Test (SPNTT), and neuromuscular control of the Deep Cervical Extensors (DCE). Test-retest reliability was assessed for Postural Control (SWAY) and Pressure Pain Threshold (PPT) over tibialis anterior, infraspinatus and the C3-C4 segment.

**Results:**

Intraclass Correlation Coefficient (ICC) for intra- and inter-examiner reliability was highest for ROM (range: 0.80 to 0.94), DCE (0.75 to 0.90) and CCFT (0.63 to 0.86). JPE had the lowest ICC (0.02 to 0.66). Intra- and inter-reliability for GS and SPNTT showed kappa ranging from 0.66 to 0.92, and 0.57 to 0.78 (prevalence adjusted), respectively. For the test-retest study, ICC was 0.83 to 0.89 for PPT and 0.39 to 0.79 for SWAY. Construct validity was satisfactory for all tests, except JPE. Significant between group discriminative validity was found for CCFT, ROM, GS, SPNTT and PPT, however, differences were within the limits of the minimal detectable change.

**Conclusions:**

The majority of the tests evaluated showed satisfactory reliability and construct validity supporting their use in the clinical evaluation of patients with chronic neck pain.

**Electronic supplementary material:**

The online version of this article (doi:10.1186/1471-2474-15-408) contains supplementary material, which is available to authorized users.

## Background

Musculoskeletal disorders are the most common form of long-term illness and neck pain is a frequent complaint [1]. The point prevalence of neck pain is around 20% [2, 3] and the one-year prevalence around 35% [2, 4].

People with chronic neck pain present with a number of objective findings including alterations in the structure and function of the deep cervical flexor [5, 6] and extensor muscles [7, 8], reduced range of neck motion [9], proprioceptive deficits [10, 11], occulomotor disturbances [12, 13], impaired postural control [14, 15], and general sensitization of the central nervous system [16, 17].

Several clinical tests have been described to test for these deficits, however, the reliability of such tests have not been adequately evaluated or have only been evaluated when implemented with advanced technologies which would not be available within a clinical setting. For instance, several studies conducted on people with chronic neck pain, cervicogenic headache or asymptomatic controls [18–21], have found satisfactory intra- and inter examiner reliability of the cranio-cervical flexion test, a low-load test, measuring the patient’s ability to activate the deep cervical flexor muscles. However, a systematic review concluded that the reliability of this test was under the acceptable level [22]. In contrast, no standardized clinical test has been described to test the neuromuscular control of the deep cervical extensors. Reliability of measuring repositioning error during tests of relocation accuracy has been examined in people with whiplash-induced neck pain and in asymptomatic controls with advanced equipment only, and the results are conflicting - ranging from high levels of reliability [23], to very low levels [24]. Gaze stability and the smooth pursuit neck torsion test, tests of oculomotor control, have been widely described and applied in the assessment of people with neck pain [12, 25]. The test-retest reliability of Gaze stability has been reported to be fair to good in asymptomatic controls when using wireless 3D sensors to monitor neck movement [12, 26]. Postural control has been examined extensively on a force platform, but only one study has evaluated the reliability of measuring postural control in adults in a clinical setting (using a Wii Balance Board) and this study evaluated healthy individuals only [27]. Satisfactory reliability has been reported for the measure of pressure pain threshold (PPT) using a hand-held algometer in patients with acute neck pain [28], however, this has not been replicated in patients with chronic neck pain.

Thus, although widely used clinically, very few clinical tests applied during the assessment of a person with chronic neck pain have been evaluated for their reliability.

This study, therefore, investigates intra- and inter-examiner reliability of six clinical tests – Cranio-Cervical Flexion Test (CCFT), Cervical Range of Movement (ROM), Joint Position Error (JPE), Gaze Stability (GS), Smooth Pursuit Neck Torsion Test (SPNTT), neuromuscular control of the deep cervical extensors (DCE), and test-retest reliability of postural control (SWAY) and Pressure Pain Threshold (PPT) in patients with and without chronic neck pain. As a secondary aim, the construct and between groups discriminative validity of these tests is examined.

## Methods

### Design

The study was a reproducibility study of six clinical tests with two examiners, and a test-retest study of two physical performance tests, conducted by a third examiner. The study followed a strict three-phase reproducibility protocol, including a “training”, an “overall agreement”, and a “study” phase, as recommended for nominal and ordinal data [29]. Since the clinical tests included primarily ratio interval data, the protocol was adjusted to a two-phase study by excluding the overall agreement phase [30]. This standardized protocol included a case as well as a control group, to confirm that both groups could be tested reliably. Tests were described in detail by examiner C (Experienced Physiotherapist (PT) and Manual Therapist) during phase one. Afterwards, examiner A and B (final year bachelor PT students), and examiner C tested 10 subjects with and without neck pain in an open study, to become familiar with and to standardise and equalise the test procedure and interpretation of results. During phase two, the examiners applied all tests on included subjects. Examiners were blinded to the status of the subjects, except for examiner C, since this examiner was involved in the recruitment of cases and controls. Although examiner C was aware of the subject’s status, this examiner only performed the PPT and SWAY tests, which are two fairly objective tests, thus limiting potential bias. All examiners were mutually blinded to the results of other examiners.

### Study sample

Patients were recruited at physiotherapy clinics and controls via local advertisements. *Inclusion criteria for neck pain patients:* adults (>18 years), neck pain >6 months, reduced neck function (Neck Disability Index; NDI, minimum 10/50), pain primarily in the neck, and ability to read and understand Danish.

*Inclusion criteria for controls:* no present pain in neck, shoulder, elbow or hand, no neck pain lasting more than one week during the last year, matched on gender and age (+/-3 years to one of the patients), and ability to read and understand Danish.

**Figure 1:**
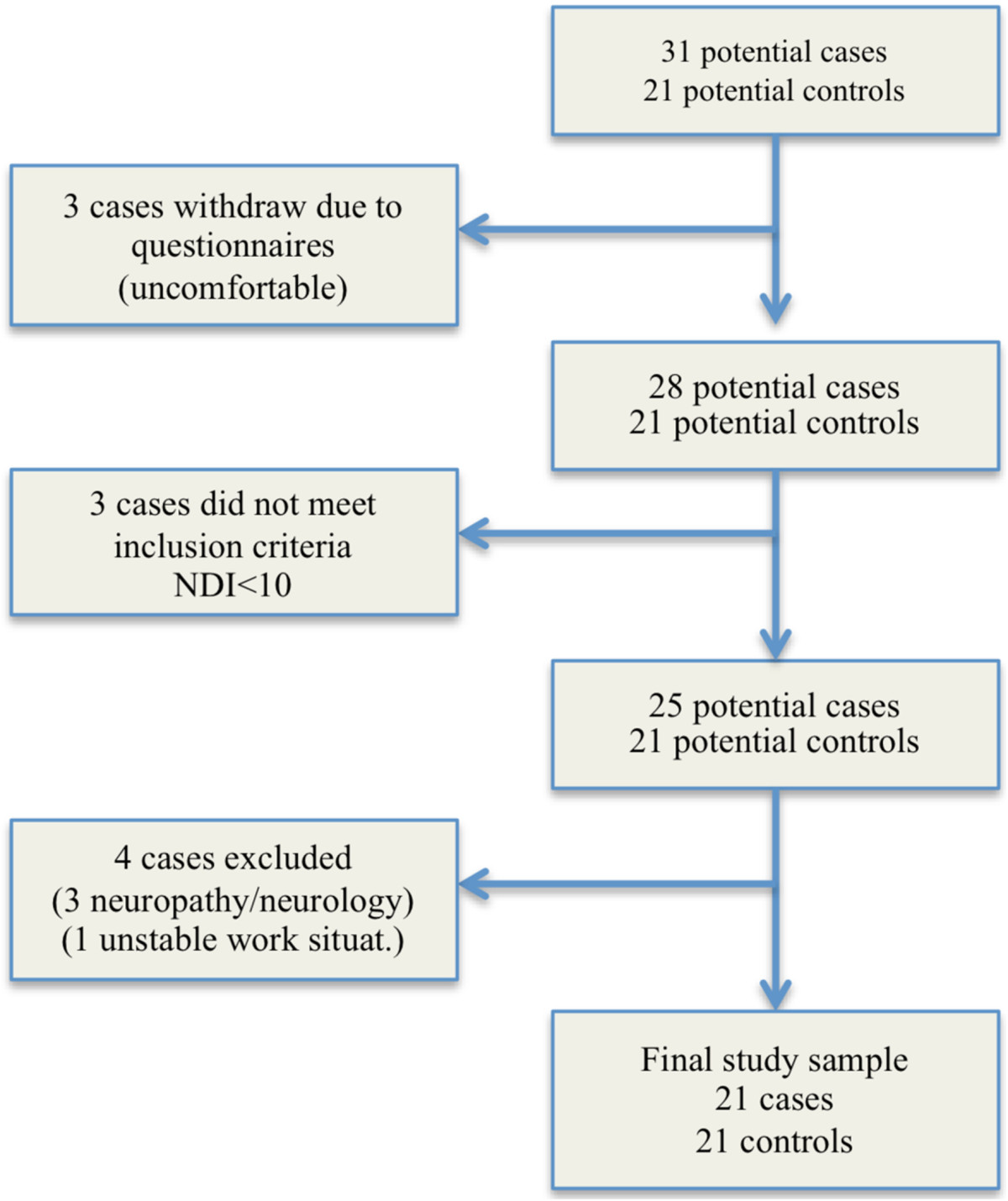
**Participant flow and retention.**

*Exclusion criteria for both groups:* neuropathies/radiculopathies (defined by positive Spurling, cervical traction and plexus brachialis tests) [31], neurological deficits, being in an unstable social and/or working situation, pregnancy, known fractures, and depression according to the Beck Depression Inventory (BDI) (score >29) [32].Overall, 31 patients with chronic neck pain were recruited, and 21 were included in the final sample (Figure 1). All 21 controls were matched on gender and age (+/-3 years). Subjects received oral and written information about the project and gave their written informed consent to participate. The Regional Scientific Ethical Committee of Southern Denmark approved the study (S-20100069). The study conformed to The Declaration of Helsinki 2008.

### Questionnaires and self-reported outcomes

Subjects completed self-reported questionnaires prior to enrolment, and demographics (age, gender, height and weight, type of accident, medication, symptom development over the last two months, employment and educational status) were registered. Questionnaires included the NDI (range: 0 to 50) [33], Medical Outcomes Study Short Form 36 (SF36)(range: 0 to100), with emphasis on the Physical Component Score (SF36-PCS) [34, 35], Numeric Rating Scale (NRS) for present pain (P.P.) and average pain during the last week (Week) [36], Modified Global Perceived Effectiveness (GPE) to evaluate stability of the condition with the question: “Compared to your first visit, how would you describe your neck today?” (-5 = vastly worse, 0 = unchanged, and 5 = completely recovered) [37]. Only subjects answering 0, representing unchanged, were included for intra- examiner and test-retest reliability.

### Clinical tests

Subjects were not allowed to practice the tests, except for the tests of neuromuscular control; CCFT and DCE. For the CCFT, subjects performed three practice trials and for DCE one practice trial for a maximum of 30s. Test instruction followed an instruction manual, however, the amount of instruction and feedback varied among subjects, depending on the subject’s ability to understand the procedure.

*Cranio Cervical Flexion Test (CCFT)* was performed, using a Pressure Biofeedback Unit (Stabilizer; Chattanooga Group, South Pacific), as described by Jull et al. [38]. The subject was asked to perform cranio-cervical flexion in five incremental stages guided by the pressure sensor. The activation score has six scoring options; 20,22,24,26,28 and 30 mmHg.

*Deep Cervical Extensor (DCE)* test was performed in prone with their head over the edge of the bed. A laser was fixed to the top of the subject’s head and was projected to a target. The duration of time the laser beam was kept within the centre of the target was measured in seconds (sec.).

**Figure 2:**
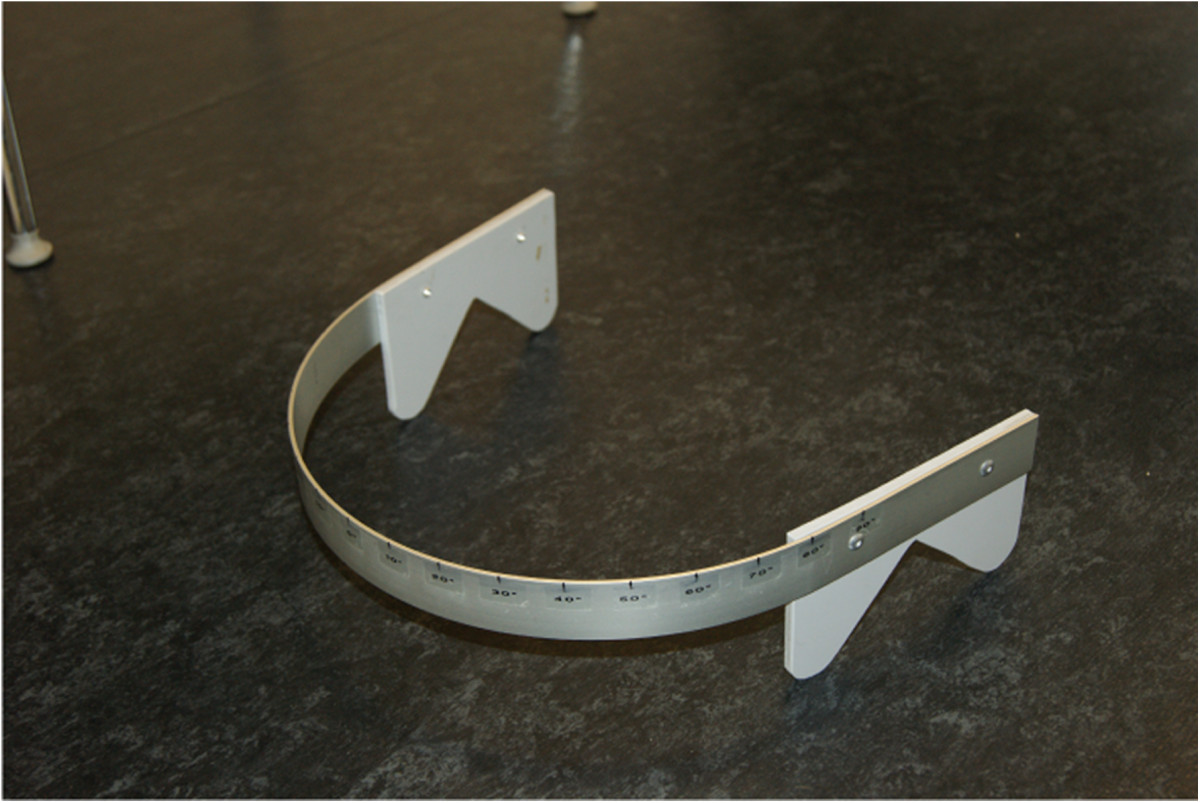
**Device for the measurement of rotation ROM.**

*Range of movement (ROM)* was examined using a bubble inclinometer (Baseline Bubble Inclinometer, Fabrication Enterprises Inc, USA) for flexion/extension and lateral flexion, and custom-made equipment for neck rotation (Figure 2). All scores were registered to the nearest degrees, except for rotation which was registered to the nearest 5 degrees.

*Joint Position Error (JPE)* JPE was examined following return from active rotation, flexion, and extension movements by measuring the reposition error. A laser beam was positioned 1 meter behind the subject, and the laser was projected to a cm ruler attached to a cap which the subjects wore. Data was registered in millimetres (mm).

*Gaze stability (GS)* was registered during rotation, flexion and extension movements as positive/negative based on the patients report of symptoms such as nausea, dizziness, disturbed vision.

*Smooth Pursuit Neck Torsion Test (SPNTT)* was tested in both a neutral head position and with the trunk rotated 45 degrees and was registered as positive/negative based on the patients report of symptoms such as nausea, dizziness, disturbed vision.

*Postural control* was measured during one-legged stance (eyes open and eyes closed) using a Wii balance board (Nintendo, Kyoto, Japan) and quantified with the SwayWithWii software program. Data was registered in millimetres (mm).

*Pressure Pain Threshold (PPT)* was examined at three sites (neck, m. infraspinatus and m. tibialis anterior) using a hand-held algometer (Wagner, FPX algometer, USA) and was registered in kilogram-force (kgf). For further test descriptions, see Table 1 and Additional file 1: Test description.

**Table 1:** **Summary of tests included in the reproducibility and test-retest study**

Test	Subject position and task	Equipment	Outcome	Reference
**CCFT**	Supine crook lying position, cervical spine in neutral. Pressure Biofeedback placed suboccipitally and inflated to 20 mmHg. Subject performs cranio-cervical flexion through five progressive stages (22-30 mmHg)	Pressure Biofeedback (Stabilizer Chattanooga Group, South Pacific)	20,22,24, 26,28,30 mmHg.	[38]
**DCE**	Prone, legs straight, arms by the side. Laser light attached to the head, aimed at a target on the floor (60 cm distant). Subject performs low cervical extension with the cranio-cervical region maintained in a neutral position (light on target).	Laser Light, Head Band, Target sheet	Seconds (0-120)	[48]
**ROM**	Sitting, feet supported. Bubble inclinometer placed on the highest point of the head. Active flexion, extension and lateral flexion are performed. Rotation is performed with the designated equipment (Figure 2).	Bubble inclinometer (Baseline Bubble Inclinometer, Fabrication Enterprises Inc, USA)	Degrees (0-100)	[65]
**JPE**	Sitting, feet supported. Wearing a cap with a centimetre measure attached to the back (horizontal and vertical). Laser light placed behind, with starting position at 0. Active neck rotation, flexion, extension are performed with eyes closed and reposition error is measured.	Laser light, cap with millimetre measure	Millimetre (0-50)	[23]
**GS**	Sitting, feet supported. Black marker on a wall positioned 1 m in front. Subject keeps gaze fixed on the marker while performing active neck rotation, flexion and extension.	Marker	Positive/Negative based on patients report of symptoms such as nausea, dizziness, disturbed vision.	[12, 13]
**SPNT**	Sitting, feet supported. Head in neutral position. Subject follows with eyes, a pen moving horizontally from side to side ~30 cm in front whilst keeping head still. Task repeated with the neck in torsion position	Marker, Pen	Positive/Negative based on patients report of symptoms such as nausea, dizziness, disturbed vision.	[57]
**SWAY**	1) Standing, feet together. Arms crossed over chest. Keep focus on a black marker positioned 2.5 m in front, stand as still as possible (eyes open and closed). 2) Standing on the non-dominant leg. Arms crossed over chest. Keep focus on a black marker 2.5 m in front, stand as still as possible.	Wii balance board (Nintendo, Kyoto, Japan), SwayWithWii software program	95% Confidence Ellipse Area	[63]
			Anterior/Posterior sway (cm.)	
			Medio/Lateral sway (cm.)	
			Centre of Pressure Path Length (cm.)	
**PPT**	Supine for PPT over tibialis anterior. Prone for PPT over the infraspinatus and over the cervical column, between the transverse processes of C3 and C4. Pressure applied at a slow rate.	Hand-held algometer (Wagner, FPX algometer, USA)	Kgf	[28]

Tests included were Cranio-Cervical Flexion Test (CCFT), Deep Cervical Extensor test (DCE), Range of Movement (ROM), Joint Position Error (JPE), Gaze Stability (GS), Smooth Pursuit Neck Torsion Test (SPNTT), Balance/Postural Control (SWAY) and Pressure Pain Threshold (PPT).

The pictures were produced for the purposes of this study. The subjects appearing in the pictures have provided consent to publish.

### Procedure

**Figure 3:**
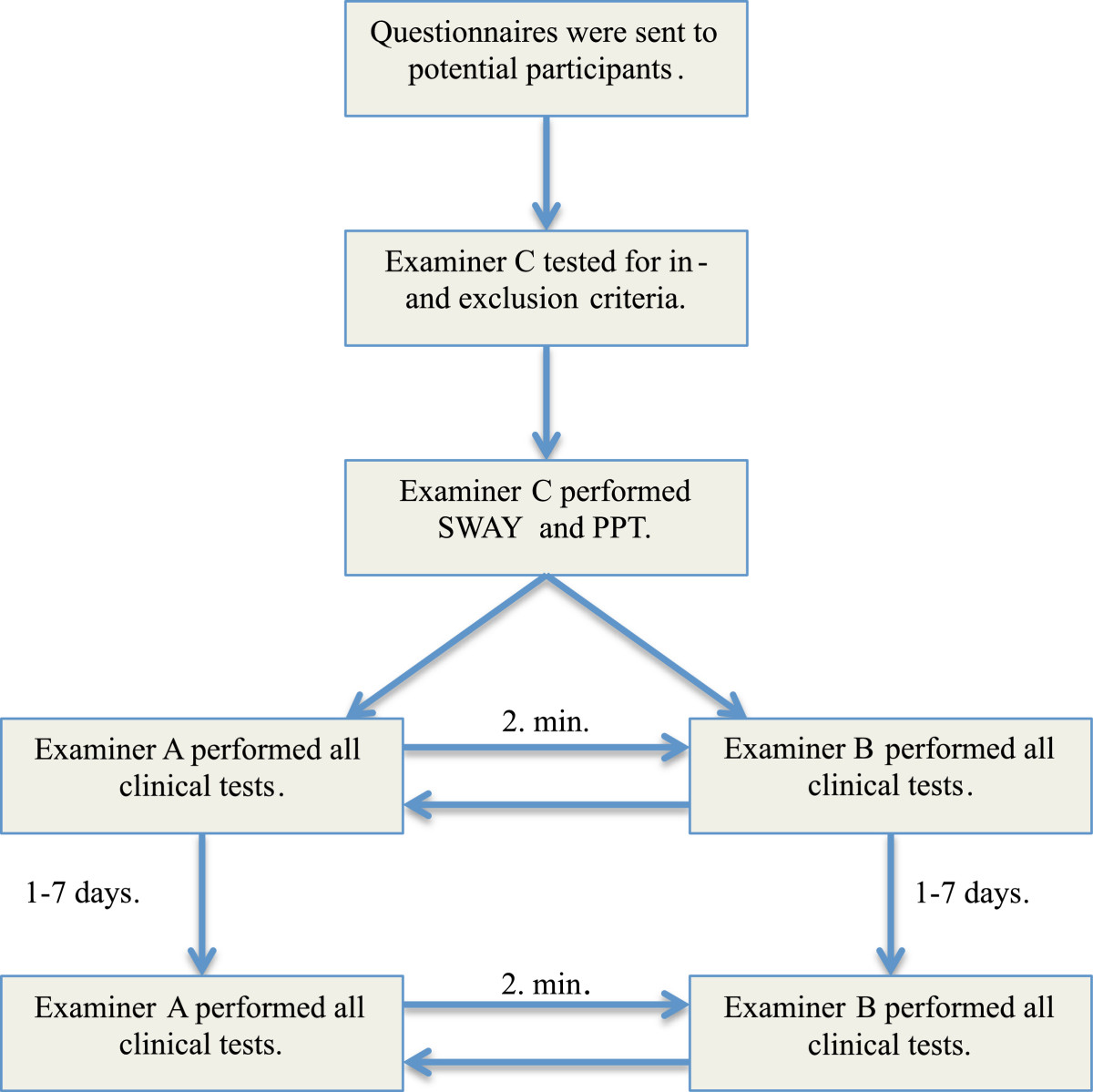
**Test procedure.**

Questionnaires were sent to participants before their first appointment. At the first visit, participants reported NRS (P.P./Week). Examiner C then screened for in- and exclusion criteria, after which SWAY and PPT tests were conducted. Following a rest period of ~2 min., tests for intra- and inter-reliability were performed. Testing order of examiner A and B was randomized for the first test round, and test order was always CCFT, ROM, JPE, GS, SPNTT and DCE. After a rest period of ~2 min. the other examiner performed the same tests in the same order on the same subjects (Figure 3). Duration between the two test occasions was 1–7 days. At the second visit GPE was added, and test order of examiner A and B was reversed. Cases and controls followed the same procedure throughout the testing session.

### Sample size

Sample size was calculated based on the JPE test [23], since larger standard deviations were expected for this test. Sample size was estimated based on the 95% confidence interval according to the recommendation from Hopkins [39]. In a two one-sided test analysis for additive equivalence of paired means with bounds -5 and +5 for the mean difference and a significance level of 0.05, assuming a mean difference of 0, a common standard deviation of 16 and correlation 0.9, a sample size of 19 pairs, was required to obtain a power of at least 0.8.

### Statistical analysis

Data analysis was performed blinded. Summary statistics are based on whole group-mean scores from examiner A and examiner B at the first test occasion. For the test-retest study, whole group-means are based on the first test examination. Mean values from the three repetitions for DCE, JPE, PPT and SWAY was used for analysis of reproducibility.

For calculation of intra- and inter-examiner reproducibility for ratio interval data, ICC (2,1) and Bland and Altman’s with 95% limits of agreement (LOA) were used. Interpretation of ICC was 1.00-0.76 (good to excellent), 0.75-0.41 (fair to good), and 0.40-0.00 (poor) [40]. The minimal detectable change (MDC) that is not due to error was calculated for all parametric tests, as 1.96 * √2 * SEM [41]. The standard error of measurement (SEM) was calculated as SEM = standard deviation of the mean difference between tester A and B divided by √2 [42].

For ordinal data, Cohen’s κ statistics with 95% confidence interval were calculated, with the interpretation 1.00-0.81 (almost perfect), 0.61-0.80 (substantial), 0.41-0.60 (moderate), 0.21-0.40 (fair), 0.00-0.20 (slight) and below 0.00 (poor) [43]. Furthermore, observed agreement, prevalence and expected agreement were calculated. Prevalence of the index condition was calculated as $\frac{(a+\left(b+c\right)/2}{n}$. The prevalence-adjusted-bias-adjusted kappa (PABAK) was calculated for the SPNTT, in which the values in cells a and d from the contingency table are replaced with the mean values from these cells, and values from cells b and c are replaced with the mean values from these cells [44]. Whole group results will be displayed if there is no systematic bias for cases or controls.

Construct validity between each of the clinical tests and NDI, NRS and SF36 were calculated using Spearman’s correlation coefficient (rho), due to non-normal distributions. Correlations were interpreted as for the ICC: 1.00-0.76 (strong), 0.75-0.41 (moderate), and 0.40-0.00 (weak). Positive correlation coefficients indicate positive associations, negative indicate negative associations. Since interpretation from the spearman is known to be difficult, statistical significance testing was included. Between-groups discriminative validity of the clinical tests was evaluated by a t-test and a Mann–Whitney U-test in normally and non-normally distributed data, respectively. Calculations of construct and discriminative validity were based on mean scores from the first test occasion. STATA statistical software was used for all analyses (Stata Corp., 2000, College Station, TX).

## Results

A total of 42 subjects (age: 45.0 ± 15.6 years) were recruited, with 21 in each group. The groups did not differ in demographics (age, gender, height or weight). The patient group (cases) reported higher scores on the NDI (p < 0.01) and BDI (p < 0.01), lower scores on the PCS (p < 0.01) and Mental Component Score of the SF36 (SF36-MCS) (p < 0.01), compared to controls (Table 2). Eight cases had whiplash-induced neck pain and 13 had idiopathic neck pain. A total of 11 cases and 16 controls had a GPE of 0 and were included for intra- examiner and test-retest reliability (see Table 3 for the summary statistics of clinical tests.).

**Table 2:** **Self-reported demographic data for cases and controls**

Variable	Cases			Controls			
	(n = 21)			(n = 21)			
	Mean	SD	Range	Mean	SD	Range	p-value
**Age (years)**	46.1	15.6	(23–77)	44.7	14.8	(22–71)	0.76
**Height (cm)**	168.6	7.5	(157–185)	172	6.0	(160–185)	0.12
**Weight (kg)**	68.1	10.3	(52–88)	66.2	9.1	(56–88)	0.53
	**Mean**	**Median**	**Range**	**Mean**	**Median**	**Range**	
**NDI**	15.3	14	(11–27)	1.9	2	(0–4)	<0.01
**BDI**	5.7	5	(0–23)	2.2	0	(0–14)	<0.01
**SF36-PCS**	42.1	41.1	(29–64)	53.8	55.5	(39–62)	<0.01
**SF36-MCS**	50.4	50.5	(25–93)	57.6	59.1	(38–68)	<0.01
**NRS (P.P.)**	4.4	4	(1–8)	------	------	------	------
**NRS (Week)**	5.1	6	(2–10)	------	------	------	------
**Pain duration (months)**	92.8	30	(6–420)	------	------	------	------

Self reported data on age, height, weight, Neck Disability Index (NDI), Beck Depression Inventory (BDI), Physical Component Score of SF36-PCS, Mental Component Score of the SF36-MCS, Present Pain (P.P.) intensity and average pain during last week (Week) on a Numeric Rating Scale (NRS).

------ Information from controls not gathered.

**Table 3:** **Summary statistics (mean, median, SD and Range) of clinical and performance tests**

Test	Variable	Mean	Median	SD	Range
**CCFT** (mmHg.)	CCFT	24.1	23.5	2.5	(20–30)
**DCE** (sec.)	Deep cervical extensors	65.6	71.0	41.4	(3–120)
**ROM** (degrees)	Flexion	44.3	46.0	12.9	(18–70)
	Extension	55.1	57.3	15.1	(15–90)
	Left rotation	70.8	72.5	14.2	(20–95)
	Right rotation	72.4	75.0	13.2	(25–90)
	Left lateral flexion	34.8	35.0	7.5	(17–48)
	Right lateral flexion	35.9	34.8	7.9	(17–54)
**JPE** (mm.)	JPE flexion	5.9	4.7	3.8	(0–16)
	JPE extension	5.8	5.5	3.4	(0–16)
	JPE left rotation	5.4	4.4	3.5	(0–15)
	JPE right rotation	5.2	4.3	2.8	(0–16)
**SWAY** (cm.)	Romberg eyes open (CEA)	5.3	4.7	3.7	(1.2-18.3)
	Romberg eyes open (A/P)	2.7	2.6	0.9	(1.0-5.0)
	Romberg eyes open (M/L)	2.5	2.6	0.7	(1.2-4.3)
	Romberg eyes open (COP)	4.8	4.7	1.1	(2.7-6.5)
	Romberg eyes closed (CEA)	8.0	7.3	4.0	(2.7-20.6)
	Romberg eyes closed (A/P)	3.2	3.2	0.9	(2.0-6.5)
	Romberg eyes closed (M/L)	3.5	3.4	0.9	(1.9-5.6)
	Romberg eyes closed (COP)	7.2	7.1	2.0	(4.0; 13.3)
	Singe leg stance (CEA)	9.6	9.4	4.1	(2.9-22.4)
	Singe leg stance (A/P)	4.2	4.1	1.1	(2.3-6.6)
	Singe leg stance (M/L)	3.3	3.4	0.7	(1.7-5.6)
	Singe leg stance (COP)	12.3	12.4	3.1	(6.6-17.4)
**PPT (kgf)**	PPT Tibialis Anterior	4.0	3.6	1.6	(1.4-6.9)
	PPT C3-C4	2.3	2.1	1.0	(0.6-5.1)
	PPT Infraspinatus	3.1	2.7	1.5	(1.1-7.3)

Confidence Ellipse Areal (CEA), Anterior/Posterior displacement (A/P), Medial/Lateral displacement (M/L), Centre of Pressure path length (COP).

Bland Altman plots revealed that differences between examiners did not depend systematically on mean score for any of the tests, but LOA were generally wide. Highest ICC for clinical tests were found for ROM (ICC: 0.80 to 0.93), DCE (0.75 to 0.90) and CCFT (0.63 to 0.86) and lowest for JPE (0.02 to 0.66) (Table 4). Intra- and inter-reliability for GS and SPNTT showed kappa ranging from 0.66 to 0.92, and 0.57 to 0.78 (prevalence adjusted), respectively (Table 5). Overall agreement and κ-values were generally high for GS and SPNTT. PABAK calculation for SPNTT (low prevalence) increased kappa from 0.46 and 0.74, to 0.57 and 0.78 (Table 5). In the test-retest study of performance tests highest reliability was obtained for PPT (ICC: 0.83 to 0.89) compared to SWAY (0.39 to 0.79) (Table 6).

**Table 4:** **Intra- and inter examiner reliability of Cranio-Cervical Flexion Test (CCFT), Deep Cervical Extensor test (DCE), Range of Movement (ROM) and Joint Position Error (JPE)**

Test	Variable	N	MDC	ICC	(95% CI)	95% LOA
**CCFT** (mmHg.)	***Inter-reliability 1***	42	4.70	0.63	(0.41; 0.78)	(-4.99; 4.42)
	***Inter-reliability 2***	36	3.12	0.82	(0.67; 0.91)	(-3.11; 3.11)
	***Intra-reliability A***	27	2.94	0.86	(0.72; 0.93)	(-3.17; 2.72)
	***Intra-reliability B***	27	3.99	0.70	(0.43; 0.85)	(-3.92; 4.07)
**DCE** (sec.)	***Inter-reliability 1***	36	58.01	0.76	(0.59; 0.86)	(-53.24; 62.78)
	***Inter-reliability 2***	29	57.36	0.75	(0.55; 0.87)	(-67.72; 47.00)
	***Intra-reliability A***	26	37.25	0.90	(0.79; 0.95)	(-31.21; 43.29)
	***Intra-reliability B***	25	59.33	0.77	(0.55; 0.89)	(-65.86; 52.79)
**ROM** (degrees)	***Inter-reliability 1***					
	Flexion	42	14.65	0.82	(0.66; 0.91)	(-11.29; 18.00)
	Extension	42	18.90	0.80	(0.62; 0.90)	(-23.50; 14.31)
	Left rotation	42	11.21	0.92	(0.86; 0.96)	(-10.73; 11.68)
	Right rotation	42	10.11	0.93	(0.87; 0.96)	(-10.58; 9.63)
	Left lateral flexion	42	8.31	0.85	(0.74; 0.92)	(-8.22; 8.41)
	Right lateral flexion	42	9.30	0.84	(0.72; 0.91)	(-9.11; 9.49)
**ROM**	***Inter-reliability 2***					
	Flexion	36	15.02	0.83	(0.70; 0.91)	(-13.72; 16.33)
	Extension	36	12.60	0.92	(0.85; 0.96)	(-14.13; 11.07)
	Left rotation	36	9.83	0.94	(0.89; 0.97)	(-11.20; 8.44)
	Right rotation	36	10.73	0.93	(0.86; 0.96)	(-10.74; 10.74)
	Left lateral flexion	36	9.35	0.83	(0.66; 0.91)	(-7.40; 11.29)
	Right lateral flexion	36	10.06	0.80	(0.61; 0.90)	(-7.93; 10.70)
**ROM**	***Intra-reliability A***					
	Flexion	27	17.00	0.81	(0.62; 0.91)	(-13.78; 20.23)
	Extension	27	21.23	0.81	(0.63; 0.91)	(-22.60; 19.86)
	Left rotation	27	10.98	0.90	(0.75; 0.95)	(-8.21; 13.76)
	Right rotation	27	10.64	0.89	(0.76; 0.92)	(-8.42; 12.87)
	Left lateral flexion	27	8.70	0.85	(0.69, 0.93)	(-8.51; 8.88)
	Right lateral flexion	27	9.48	0.84	(0.68; 0.92)	(-10.26; 8.71)
**ROM**	**Intra-reliability B**					
	Flexion	27	14.24	0.86	(0.71; 0.93)	(-13.87; 20.23)
	Extension	27	18.91	0.82	(0.64; 0.91)	(-15.91; 21.91)
	Left rotation	27	13.05	0.87	(0.73; 0.94)	(-12.12; 13.97)
	Right rotation	27	11.00	0.88	(0.74; 0.95)	(-8.59; 13.40)
	Left lateral flexion	27	8.92	0.84	(0.68; 0.92)	(-7.44; 10.40)
	Right lateral flexion	27	9.12	0.85	(0.70; 0.93)	(-8.23; 10.01)
**JPE**	***Inter-reliability 1***					
	Flexion	42	7.59	0.54	(0.26, 0.73)	(-5.74; 9.44)
	Extension	42	9.26	0.35	(0.06; 0.59)	(-8.60; 9.92)
	Left rotation	42	6.99	0.58	(0.34; 0.75)	(-5.91; 8.07)
	Right rotation	42	8.32	0.27	(-0.02; 0.52)	(-7.25; 9.39)
**JPE**	***Inter-reliability 2***					
	Flexion	35	9.14	0.11	(-0.20; 0.41)	(-7.63; 10.65)
	Extension	35	8.22	0.36	(0.02; 0.62)	(-8.11, 8.34)
	Left rotation	35	7.97	0.02	(-0.30; 0.33)	(-6.82; 9.12)
	Right rotation	35	9.10	0.04	(-0.29; 0.37)	(-8.28; 9.92)
**JPE**	***Intra-reliability A***					
	Flexion	27	7.71	0.42	(0.05; 0.69)	(-7.48; 7.16)
	Extension	27	7.58	0.50	(0.16; 0.74)	(-7.29, 8.15)
	Left rotation	27	7.54	0.18	(-0.20; 0.52)	(-8.06; 6.64)
	Right rotation	27	9.95	0.05	(-0.36; 0.42)	(-10.36;10.55)
**JPE**	***Intra-reliability B***					
	Flexion	27	10.12	0.23	(-0.12; 0.55)	(-10.84; 9.16)
	Extension	27	8.35	0.50	(0.15; 0.74)	(-8.11; 7.79)
	Left rotation	27	5.76	0.66	(0.37; 0.83)	(-7.92; 5.86)
	Right rotation	27	6.16	0.35	(-0.03; 0.65)	(-6.31; 6.14)

Minimal Detectable Change (MDC), Intraclass Correlation Coefficient (ICC), with 95% Confidence Intervals (95% CI), and 95% Limits of Agreement (LOA).

Inter-reliability 1. test occasion (Inter-reliability 1), Inter-reliability 2. test occasion (Inter-reliability 2), Intra-reliability examiner A (Intra-reliability A), Intra-reliability examiner B (Intra-reliability B).

**Table 5:** **Inter- and intra examiner reliability of Gaze Stability (GS) and Smooth Pursuit Neck Torsion Test (SPNTT)**

Variable		N	Prevalence of index condition	Expected agreement (%)	Overall agreement (%)	κ -value	PAB-AK	(95% CI)
**GS**	Inter 1	42	0.48	50.00	85.71	0.71	-	(0.50; 0.93)
	Inter 2	36	0.56	50.46	83.33	0.66	-	(0.42; 0.91)
	Intra A	27	0.43	51.03	96.30	0.92	-	(0.78; 1.00)
	Intra B	27	0.50	49.93	88.89	0.78	-	(0.54; 1.00)
**SPNTT**	Inter 1	42	0.27	59.98	78.57	0.46	0.57	(0.17; 0.76)
	Inter 2	36	0.38	52.78	86.11	0.71	0.72	(0.47; 0.94)
	Intra A	27	0.22	64.33	85.19	0.59	0.70	(0.24; 0.93)
	Intra B	27	0.31	56.79	88.89	0.74	0.78	(0.47; 1.00)

κ statistics, prevalence of the index condition, expected agreement, overall agreement, Prevalence Adjusted Bias Adjusted Kappa (PABAK) and 95% Confidence Interval (95% CI).

**Table 6:** **Test-retest reliability of Pressure Pain Threshold (PPT) and Balance/Postural Control (SWAY)**

	Variable	N	MDC	ICC	(95% CI)	95% LOA
**SWAY** (cm.)	Romberg eyes open (CEA)	27	7.91	0.52	(0.18; 0.75)	(-7.42; 8.40)
	Romberg eyes open (A/P)	27	1.84	0.57	(0.24; 0.78)	(-1.87; 1.81)
	Romberg eyes open (M/L)	27	1.68	0.39	(0.03; 0.67)	(-1.50; 1.86)
	Romberg eyes open (COP)	25	1.38	0.79	(0.57; 0.90)	(-1.38; 1.40)
	Romberg eyes closed (CEA)	27	6.47	0.79	(0.59; 0.90)	(-6.26; 6.69)
	Romberg eyes closed (A/P)	27	1.96	0.60	(0.29; 0.80)	(-2.02; 1.98)
	Romberg eyes closed (M/L)	27	1.71	0.69	(0.43; 0.85)	(-1.71; 1.72)
	Romberg eyes closed (COP)	25	3.84	0.77	(0.55; 0.89)	(-2.82; 2.83)
	Single leg stance (CEA)	21	7.80	0.58	(0.21; 0.80)	(-8.54; 7.04)
	Single leg stance (A/P)	21	1.94	0.57	(0.18; 0.80)	(-1.97; 1.92)
	Single leg stance (M/L)	21	1.39	0.53	(0.14; 0.78)	(-1.51; 1.27)
	Single leg stance (COP)	21	4.74	0.75	(0.45; 0.89)	(-4.70; 4.78)
**PPT** (kgf)	Tibialis Anterior	27	1.90	0.86	(0.71; 0.91)	(-1.91; 1.88)
	C3-C4	27	0.90	0.89	(0.78; 0.95)	(-0.80; 1.01)
	Infraspinatus	27	1.65	0.83	(0.66; 0.92)	(-1.65; 1.64)

Minimal Detectable Change (MDC), Intraclass Correlation Coefficient (ICC), with 95% Confidence Intervals (95% CI) and 95% Limits of Agreement (LOA).

Confidence Ellipse Areal (CEA), Anterior/Posterior displacement (A/P), Medial/Lateral displacement (M/L), Centre of Pressure path length (COP).

All tests, except for JPE, and to some extent SWAY, correlated significantly with self-reported variables of NRS, NDI and SF36-PCS (Table 7). CCFT, ROM, GS, SPNTT and PPT showed significant between group differences; however, all differences were within the limits of MDC (Table 8).

**Table 7:** **Construct validity of clinical and performance tests**

Variable (n = 42)		NDI		SF36-PCS		NRS (P.P)	
		Spearman (rho)	p-value	Spearman (rho)	p-value	Spearman (rho)	p-value
**CCFT**		-0.40	<0.01	0.56	<0.01	-0.37	0.02
**ROM**	Flexion	-0.47	<0.01	0.31	0.04	-0.46	<0.01
	Extension	-0.65	<0.01	0.46	<0.01	-0.74	<0.01
	Left rotation	-0.47	<0.01	0.46	<0.01	-0.51	<0.01
	Right rotation	-0.54	<0.01	0.47	<0.01	-0.53	<0.01
	Left lateral flexion	-0.64	<0.01	0.46	<0.01	-0.67	<0.01
	Right lateral flexion	-0.62	<0.01	0.45	<0.01	-0.66	<0.01
**JPE**	Flexion	-0.05	0.75	-0.17	0.27	-0.02	0.90
	Extension	-0.04	0.82	0.07	0.65	-0.13	0.40
	Left rotation	0.25	0.11	-0.20	0.20	0.20	0.20
	Right rotation	0.23	0.14	-0.11	0.51	0.15	0.36
**DCE**		-0.33	0.047	0.14	0.41	-0.36	0.03
**SWAY**	Romberg EO (CEA)	0.12	0.45	-0.26	0.09	0.04	0.80
	Romberg EO (A/P)	0.11	0.49	-0.26	0.10	0.08	0.63
	Romberg EO (M/L)	0.18	0.26	-0.31	0.05	0.13	0.41
	Romberg EO (COP)	-0.02	0.92	-0.34	0.09	-0.04	0.85
	Romberg EC (CEA)	0.38	0.01	-0.49	<0.01	0.33	0.04
	Romberg EC (A/P)	0.40	<0.01	-0.49	<0.01	0.35	0.02
	Romberg EC (M/L)	0.31	0.05	-0.39	0.01	0.24	0.13
	Romberg EC (COP)	0.09	0.66	-0.37	0.07	0.21	0.31
	SLS (CEA)*	0.35	0.04	0.33	0.06	0.39	0.02
	SLS (A/P)*	0.38	0.02	-0.37	0.03	0.36	0.04
	SLS (M/L)*	0.29	0.09	-0.32	0.06	0.32	0.06
	SLS (COP)*	-0.14	0.57	-0.26	0.28	-0.06	0.80
**PPT**	Tibialis anterior	-0.28	0.08	0.13	0.42	-0.34	0.03
	C3-C4	-0.41	<0.01	0.31	0.05	-0.37	0.02
	Infraspinatus	-0.33	0.03	0.25	0.11	-0.43	<0.01
**GS**	Tester A	-0.80	<0.01	0.63	<0.01	-0.69	<0.01
	Tester B	-0.51	<0.01	0.49	<0.01	-0.42	<0.01
**SPNT**	Tester A	-0.49	<0.01	0.39	0.01	-0.55	<0.01
	Tester B	-0.52	<0.01	0.45	<0.01	-0.42	<0.01

Neck Disability Index (NDI), Physical Component Score of SF 36 (SF36-PCS), Mental Component Score of SF 36 (SF36-MCS) and Present Pain (P.P.) intensity – Numeric Rating Scale (NRS).

Romberg eyes open (Romberg EO), Romberg Eyes closed (Romberg EC), Single Leg Stance (SLS), Confidence Ellipse Areal (CEA), Centre of Pressure path length (COP), Anterior/Posterior displacement (A/P), Medial Lateral displacement (M/L).

*(n=36).

**Table 8:** **Discriminative validity of clinical and performance tests**

Variable (n = 42)		N	Mean cases	Mean controls	Mean diff.	SE	(95% CI)	p-value
**CCFT**		42	23.23	24.95	1.71	0.74	(0.22; 3.21)	0.03
**ROM**	Flexion	42	38.05	50.45	12.40	3.51	(19.50; 5.31)	<0.01
	Extension	42	44.31	65.81	21.5	3.58	(28.74; 14.26)	<0.01
	Left rotation	42	65.36	76.31	10.95	--	--	0.01
	Right rotation	42	66.90	77.86	10.95	--	--	<0.01
	Left lateral flexion	42	30.29	39.33	9.05	1.84	(12.77; 5.32)	<0.01
	Right lateral flexion	42	31.02	40.83	9.81	1.93	(13.72; 5.90)	<0.01
**JPE**	Flexion	42	5.38	6.33	-0.94	--	--	0.34
	Extension	42	5.83	5.70	0.13	--	--	0.70
	Left rotation	42	6.43	4.35	2.08	--	--	0.08
	Right rotation	42	5.87	4.60	1.28	--	--	0.21
**DCE**		42	49.42	78.63	29.21	--	--	0.06
**SWAY**	Romberg EO (CEA)	42	5.13	5.52	-0.39	1.16	(-2.73; 1.95)	0.74
	Romberg EO (A/P)	42	2.60	2.70	-0.10	0.28	(-0.67; 0.47)	0.72
	Romberg EO (M/L)	42	2.51	2.56	-0.05	0.22	(-0.50; 0.40)	0.82
	Romberg EO (COP)	42	4.54	4.93	-0.39	0.42	(-1.27; 0.484	0.36
	Romberg EC (CEA)	42	9.11	6.78	2.32	1.19	(-0.70; 4.73)	0.06
	Romberg EC (A/P)	42	3.44	2.98	0.46	0.27	(-0.09; 0.99)	0.10
	Romberg EC (M/L)	42	3.69	3.33	0.36	0.28	(-0.20; 0.92)	0.20
	Romberg EC (COP)	42	7.56	6.93	0.63	0.82	(-1.07; 2.33)	0.45
	SLS (CEA)	34	10.33	8.74	1.58	1.42	(1.30; 4.47)	0.27
	SLS (A/P)	34	4.47	3.81	0.66	0.35	(-0.07; 1.38)	0.07
	SLS (M/L)	34	3.38	3.18	0.21	0.24	(-0.28; 0.69)	0.40
	SLS (COP)	34	11.81	12.72	-0.91	1.46	(-3.99; 2.18)	0.54
**PPT**	Tibialis Anterior	42	3.65	4.42	0.77	--	--	0.07
	C3-C4	42	1.84	2.69	0.85	--	--	0.03
	Infraspinatus	42	2.42	3.61	1.20	--	--	0.02
**GS**	Pearson chi2 = 21.62, p = <0.01							
**SPNT**	Pearson chi2 = 13.13, p = <0.01							

Mean and mean differences (mean diff), Standard Error (SE) and 95% Confidence Interval (95% CI).

Romberg eyes open (Romberg EO), Romberg Eyes closed (Romberg EC), Single Leg Stance (SLS), Confidence Ellipse Areal (CEA), Centre of Pressure path length (COP), Anterior/Posterior displacement (A/P), Medial Lateral displacement (M/L).

## Discussion

This study evaluated the reliability of clinical and performance tests commonly applied in the assessment of individuals with neck pain disorders, in a group of people with neck pain and a group of age and gender matched asymptomatic volunteers. Bland Altman plots revealed no systematic bias for any of the tests, but LOA were generally wide, with high MDC for most tests, indicating a relatively high degree of inherent variability. Highest ICC values were found for ROM and PPT variables, and lowest for the JPE variables. High MDC values were found for most tests, indicating a relatively high degree of inherent variability. Overall agreement and κ-values were generally high for GS and SPNTT. All tests, except for JPE, correlated significantly with at least one of the self-reported variables, meaning that poor clinical values correlated with subjective responses of poor conditions. However, the mean differences between cases and controls fell within the respective MDC on all tests.

### Cranio-cervical flexion test

Bland Altman plots revealed no systematic differences depending on mean scores. The intra- and inter-reliability for the CCFT was between “fair to good” and “good to excellent” (ICC: 0.63 to 0.86), in line with previous studies [45, 46]. Studies of reliability on the CCFT in asymptomatic subjects have reported slightly higher ICC values, ranging from 0.81 to 0.98 [18, 20, 21]. Studies including both symptomatic and asymptomatic populations usually have higher within subject- and day-to-day variation. The relatively large LOA and MDC, (intra-examiner: 2.9 and 3.9 mmHg; inter-examiner: 3.1 and 4.7 mmHg) (Table 4), is a future challenge for interventions. An MDC of two target levels (4.0 mmHg) is considered to be insufficient in a test with only five target levels. However, the significant correlation between CCFT and NDI, SF36-PCS and NRS, makes the test clinically relevant. The test needs improved psychometric properties for clinical use if implemented as in the present study. However, it should be noted that scoring of the CCFT may also include a measurement of endurance, that is, the number of 10 seconds holds that the subject can do at their achieved pressure level to generate a performance index. For example if a patient can achieve the second level of the test (24 mmHg) and perform six, 10 seconds holds with the correct action of cranio-cervical flexion, their performance index is 4 × 6 = 24. Highest activation score is 10 and highest performance index 100. Different results may have been achieved with this scoring approach.

### Deep cervical extensors

Bland Altman plots revealed no systematic differences depending on mean scores. ICC for the intra- and inter-examiner reliability measures ranged from 0.75 to 0.90 (“good to excellent”). This is the first study to examine the reliability of this test. Although the results are promising, the large LOA and MDC, ranging from 37 to 59 seconds, indicate higher variation than expected from the ICC. The high ICC is probably due to large between subject variability, thus disguising large test-retest differences [47]. Although testing in a cranio-cervical neutral position, as recommended for the deep neck extensors [48, 49], the validity of this test has not been confirmed. Lower scores on the test correlated with higher levels of pain (NRS) and disability (NDI), but not with SF36. The present DCE test needs improved psychometric properties for clinical use.

### Range of movement

Bland Altman plots revealed no systematic differences depending on mean scores. ICC measures for the intra- and inter-examiner reliability ranged from 0.80 to 0.93 (“good to excellent”), in line with previous studies using an inclinometer [50–52]. Since, “good to excellent” reproducibility was obtained using the custom-made rotation device; future clinical use of this device seems promising. LOA and MDC were large for neck flexion and extension (13 to 21°), reflecting some variation. Significant correlation was found between ROM variables and NDI, SF36-PCS and NRS. This test has satisfactory psychometric properties and can be recommended for clinical use.

### Joint position error

Bland Altman plots revealed no systematic differences depending on mean scores. ICC for the intra- and inter-examiner reliability measures ranged from 0.02 (“poor”) to 0.52 (“fair to good”). Previous studies have reported varying results, however, most studies report ICC above 0.75 [23, 46, 53–55]. In this study the laser light was positioned behind the subject whilst in most previous studies the laser light was attached to the head of the subject, which may explain such differences. Other explanations for differences in results could be the equipment used, or the current number of three repetitions performed, since six repetitions are recommended for stable estimates and higher reliability [53]. This was further supported by the fact that other studies using three repetitions have reported ICC similar/lower than the present study [24, 56]. JPE revealed large LOA and MDC ranging from approximately 7 to 10 mm, and did not reach significant correlations with any of the self-reported outcome measures. As described, this current test cannot be recommended for further use.

### Gaze stability

GS κ-values ranged from 0.66 (“substantial”) to 0.92 (“almost perfect”) for intra- and inter-reliability. This is the first study to examine reproducibility of the GS test in a clinical setting without sophisticated equipment. However, the present results are in line with previous studies using advanced equipment, such as wireless 3D sensors with ICC ranging from 0.40 to 0.89 [12, 26]. GS discriminated significantly between cases and controls, in line with other studies [12, 13]. Since the GS test showed significant correlations with NDI, SF36-PCS and NRS, this test has satisfactory psychometric properties and can be recommended for clinical use.

### Smooth pursuit neck torsion test

κ ranged from 0.46 (“moderate”) to 0.74 (“substantial”) for intra- and inter-reliability of the SPNTT. No other studies have evaluated the reproducibility of the SPNTT in a clinical setting. The κ-values obtained for SPNTT were lower than expected, probably due to the low prevalence of the condition, known to affect the κ-value [29]. Therefore, PABAK was used to adjust for this, and κ ranging from 0.70 to 0.78 and 0.57 to 0.72 was obtained for intra- and inter-reliability, respectively. The SPNTT test was able to discriminate significantly between cases and controls, as also shown in earlier studies of whiplash patients [57–60], while other studies reported no differences [25, 61, 62]. A positive test correlated with higher NDI and NRS scores, and lower SF36-PCS. The SPNTT has satisfactory psychometric properties and can be recommended for clinical use.

### SWAY

Bland Altman plots revealed no systematic differences depending on mean scores. Highest ICC values were obtained for the Romberg eyes closed condition with 0.79 (“good to excellent”) for 95% Confidence Ellipse Area (CEA), 0.60 (“fair to good”) for Anterior/Posterior (A/P), 0.69 (“fair to good”) for Medial/Lateral (M/L) and 0.77 (“good to excellent”) for Centre of Pressure path length (COP). All COP variables were above 0.75 (“good to excellent”). These results are in line with one previous study using the Wii balance board in healthy subjects reporting ICC above 0.75 [63]. The present study found no systematic bias, and significant correlations were found between NDI/SF36-PCS/NRS and area/range of displacement in the Romberg eyes closed condition and single-leg stance, but not for COP. Overall, the SWAY test has satisfactory psychometric properties and can be recommended for clinical use.

### Pressure pain threshold

Bland Altman plots revealed no systematic differences depending on mean scores. ICC was “good to excellent” for all variables (Tibialis anterior: 0.86, C3-C4: 0.89, Infraspinatus: 0.83), in concordance with previous studies on patients with acute neck pain [28, 64]. MDC was 1.90 kgf (Tibialis Anterior), 0.90 kgf (C3-C4), and 1.65 kgf (Infraspinatus), also in accordance with an earlier study using hand-held algometry [28]. Since significant correlations were found between PPT and NDI/NRS (all sites) and SF36-PCS (C3-C4), this test has satisfactory psychometric properties and can be recommended for clinical use.

### Considerations

A strength of the study is that it followed a standardized protocol, including a training phase, in which examiners were able to standardise and calibrate performance and interpretation of tests. The inclusion of a thorough training phase enabled inexperienced examiners to obtain satisfactory results. Further, by including a case as well as a control group, we demonstrated that both groups could be tested in a clinical manner reliably. Since ICC did not differ between groups, except for a minor tendency in the CCFT for neck pain subjects to have lower ICC, only the pooled data set was presented. Furthermore, primarily using quantifiable variables may have reduced variability which is usually introduced by more subjective estimates e.g. presence of co-contraction, breathing etc. It is unclear whether superior results would have been obtained if more experienced examiners were involved.

A weakness of the study may have been the duration of the test procedure. The entire test procedure had a length of approximately 1.5 hours, possibly imposing a fatigue effect. However, post-hoc analysis of data from the CCFT and DCE tests, comparing mean values for each of the tests in the order they were performed for each of the examiners, revealed that a significant fatigue effect was not present. Additionally, no significant learning effect was evident. The GPE was included in order to control for within-subject change between test occasions. Nevertheless, it cannot be entirely excluded that residual fatigue, not altering the subject’s perception, biased the results on the second test occasion.

Most patients were receiving treatment, and may have been familiar with the tests. Since one purpose of a reliability study is to examine all aspects of a clinical test, including information, instruction and position of subjects, this might have biased the data, possibly resulting in higher reliability estimates, since some of the patients may have been familiar with the tests. However, this may have decreased the possible difference between cases and controls, and thus resulting in lower estimates for discriminative validity.

Generally, interpretation of the data on discriminative validity must be performed with caution, since the sample size was not powered to estimate this. The general finding of the present study was that although significant differences were found for most variables, they were all within the MDC. From a clinical perspective, this naturally complicates the interpretation of tests, since “positive” findings may thus be attributed to measurement error. Sufficiently powered future studies are therefore needed, especially on discriminative and predictive validity, in addition to responsiveness, and establishment of relevant cut-off points for abnormality by investigating the normal variation in a healthy population.

## Conclusion

The majority of the examined clinical and performance tests were reliable and showed satisfactory construct validity. Although examiners were inexperienced with the tests, this standardised protocol showed that with training high reliability measures were obtained for most tests. Wide LOA and high MDC values were found, indicating a relatively high degree of inherent variability. All tests, except for JPE, correlated with variables such as NRS, NDI and SF36-PCS. None of the measures were able to differ significantly between groups within their respective MDC. Future challenges are to test the discriminative and predictive validity, in addition to responsiveness of each test in different patient populations.

## Electronic supplementary material

Additional file 1:Test description.(PDF 97 KB)

Below are the links to the authors’ original submitted files for images.

Authors’ original file for figure 1

Authors’ original file for figure 2

Authors’ original file for figure 3
