# Supplementary material for: Reliability, construct and discriminative validity of clinical testing in subjects with and without chronic neck pain
Source: BMC Musculoskelet Disord. 2014 Dec 4;15:408. doi: 10.1186/1471-2474-15-408 (PMC4325947; doi:10.1186/1471-2474-15-408)
Supplement: Supplementary file 1 — Additional file 1:Test description.(PDF 97 KB) [file 12891_2014_2400_MOESM1_ESM.pdf]

Additional file 1

Cranio Cervical Flexion Test (CCFT) followed the protocol described by Jull et al. (Jull et al., 2008). The subject was in supine crook lying with the cervical spine in a neutral position. The tests consists of performing cranio-cervical flexion to sequentially reach five pressure targets in 2 mmHg increments from a baseline of 20 mmHg to the final level of 30 mmHg, using feedback from an air-filled pressure sensor (Stabilizer™, Chattanooga Group Inc. USA) placed behind the neck. Initially the examiner facilitated the subject to perform cranio-cervical flexion with active assisted movement, in order to familiarize the subject with the task. The examiner identified the target level (activation score) that the subject could hold steadily for 10 s without resorting to retraction, without dominant use of the superficial neck flexor muscles, and without a quick, jerky cranio-cervical flexion movement. The activation score has six scoring options 20, 22, 24, 26, 28 and 30 mmHg.

Range of movement (ROM) was examined using a bubble inclinometer for flexion/extension and lateral flexion, and custom designed equipment for rotation. The subject was seated with their feet supported on the ground and the examiner asked the subject to sit with a straight back. The bubble inclinometer was placed on the highest point of the head, and the subject was asked to bend his/her head forward as far as possible for flexion and look up to the ceiling as far as possible for extension. Lateral flexion was measured holding the bubble inclinometer on the highest point of the head and the subject was asked to put his/her ear to the shoulder. Rotation was measured using the custom device (Figure 2) which was placed on the subject´s shoulder. The subject was asked to look as far as possible to the right/left. All ranges were registered to the nearest degree, and rotation to the nearest 5 degrees.

Joint Position Error (JPE) was examined using a laser light standing behind the subject. The subject was wearing a cap with a centimetre measurement attached (horizontally and vertically), and the laser light was placed so that the light targeted the 0 point both in the horizontal and vertical planes. The subject was sitting with their head in a neutral position and was asked to close the eyes and perform a full active cervical rotation to the left and then return to the starting position. The subject indicated verbally when they thought they had returned to the starting position, and the difference to the target 0-point was measured by the examiner as the reposition error in millimetres. This was repeated three times each in left rotation, right rotation, flexion and extension. Between each test movement, the subject's head was manually adjusted back to the original target 0-point (starting position) by the examiner. All data was registered in millimetres.

Gaze stability (GS) was examined with the subject sitting, feet supported, and facing a wall 1m distant. A black marker, 1 cm in diameter, was placed on the wall at eye level. The subject was asked to keep the gaze fixed on the marker and then rotate the head as far as possible, without losing fixation of their gaze on the marker. This was repeated three times in each movement direction of left rotation, right rotation, flexion and extension. The test was positive if the subject was not able to fixate their gaze (evaluated by the examiner), if the movement was performed very slowly to keep focus, or if the subject experienced dizziness, discomfort or blurred vision. Data was registered as positive/negative.

Smooth Pursuit Neck Torsion Test (SPNTT) was performed with the subject sitting, feet supported, with the examiner standing in front of the subject. The subject's head and trunk were in a neutral forward-facing position (neutral position). The examiner moved a pen horizontally from side to side

(three times) and the subject was asked to follow the pen with their eyes. This was performed at a distance of ~30 cm from the subject and angles were approximately 30 degrees. For the second part of the test, the head was held in a neutral position while the trunk was rotated 45° to the left or the right (neck torsion position) and the test was repeated. The test was positive if the subject was unable to follow the moving pen (evaluated by the examiner), if the subject had quick irregular eye movements, or if the subject experienced dizziness, discomfort or blurred vision any of which should have worsened in the torsioned versus neutral position. Data was registered as positive/negative.

Deep Cervical Extensors (DCE) was designed to target the deep extensors, the multifidus and semispinalis cervicis. Subjects wore a headband with a laser light attached to the top. The test was performed with the subject in prone on a plinth (60 cm height) with their legs straight and the arms positioned alongside their body. Their head was positioned just off the plinth, with C7 following the edge off the plinth. The subject was then instructed to perform cervical extension whilst keeping the cranio-cervical region in a neutral position. The examiner observed the movement and the subject was asked to stop when extension in middle or upper cervical column region occurred. This point was marked with a target on the floor, with the laser light targeting the centre (1cm in diameter). The subject was then instructed to relax for 30s and then repeat the same movement to find the same position with the laser light. When the laser was stable in the centre of the target, the subject was asked to hold the position for as long as possible with a maximum of 120 sec. duration. If the laser moved out of the centre of the target, the test was terminated and the time registered. Each subject performed the test three times with a rest of approximately 20 s between repetitions.

Sway tests (SWAY) were performed on a Wii balance board (Nintendo, Kyoto, Japan), with the use of the SwayWithWii software program. Subjects performed a double-leg narrow stance test (Romberg), eyes open (EO) and eyes closed (EC), and a single leg, eyes open, stance test. The double stance tests were performed with the subject standing feet together, heel to heel and toe to toe, and arms crossed lightly over the chest. The single stance tests were performed on the non-dominant leg with arms crossed over the chest. The subject was instructed to lift the foot from the floor and hold the foot against the medial side of the contralateral lower leg, the toes touching the medial malleolus of the opposite ankle. In the eyes open conditions, subjects were instructed to focus on a black dot placed on a wall 2.5 m in front of them. The subject was instructed to stand as still as possible for 30 s without talking. Data was registered in the software program (SwayWithWii). The double stance eyes open test was performed once, while the two other tests were performed three times. Data was registered in mm and cm.

Pressure Pain Threshold (PPT) was examined at three sites using a hand-held algometer (Wagner, Force Ten, FDX). 1) Over the tibialis anterior (TA) muscle belly, 5cm distal of the tuberositas tibia and 2cm lateral of margo anterior tibialis. 2) over the cervical column, between processus spinosus of C3-C4 and a mark 2 cm lateral to this point. 3) over the infraspinatus muscle, 3cm inferior to the spina scapula and 3cm lateral to the medial boarder of the scapula. Pressure was applied at a constant slow rate, and the subject was instructed to say stop when the pressure applied changed from pressure to pain. Measurements were repeated three times at each site. All measures were registered in kilogram-force (kgf).
