# Supplementary figures and images for: Reliability, construct and discriminative validity of clinical testing in subjects with and without chronic neck pain
Source: BMC Musculoskelet Disord. 2014 Dec 4;15:408. doi: 10.1186/1471-2474-15-408 (PMC4325947; doi:10.1186/1471-2474-15-408)

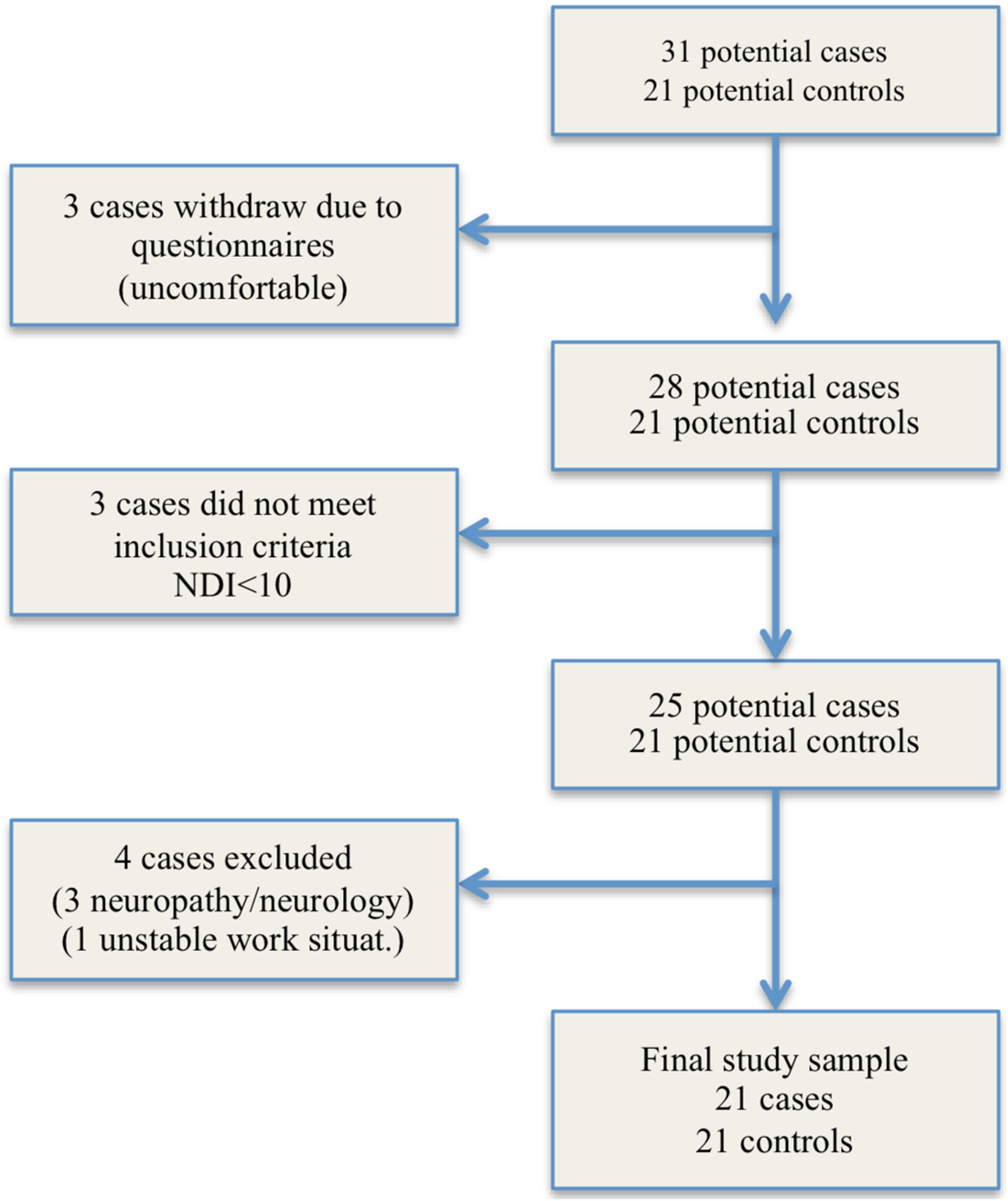

Supplement: Supplementary file 2 — Authors’ original file for figure 1 [file 12891_2014_2400_MOESM2_ESM.tif]

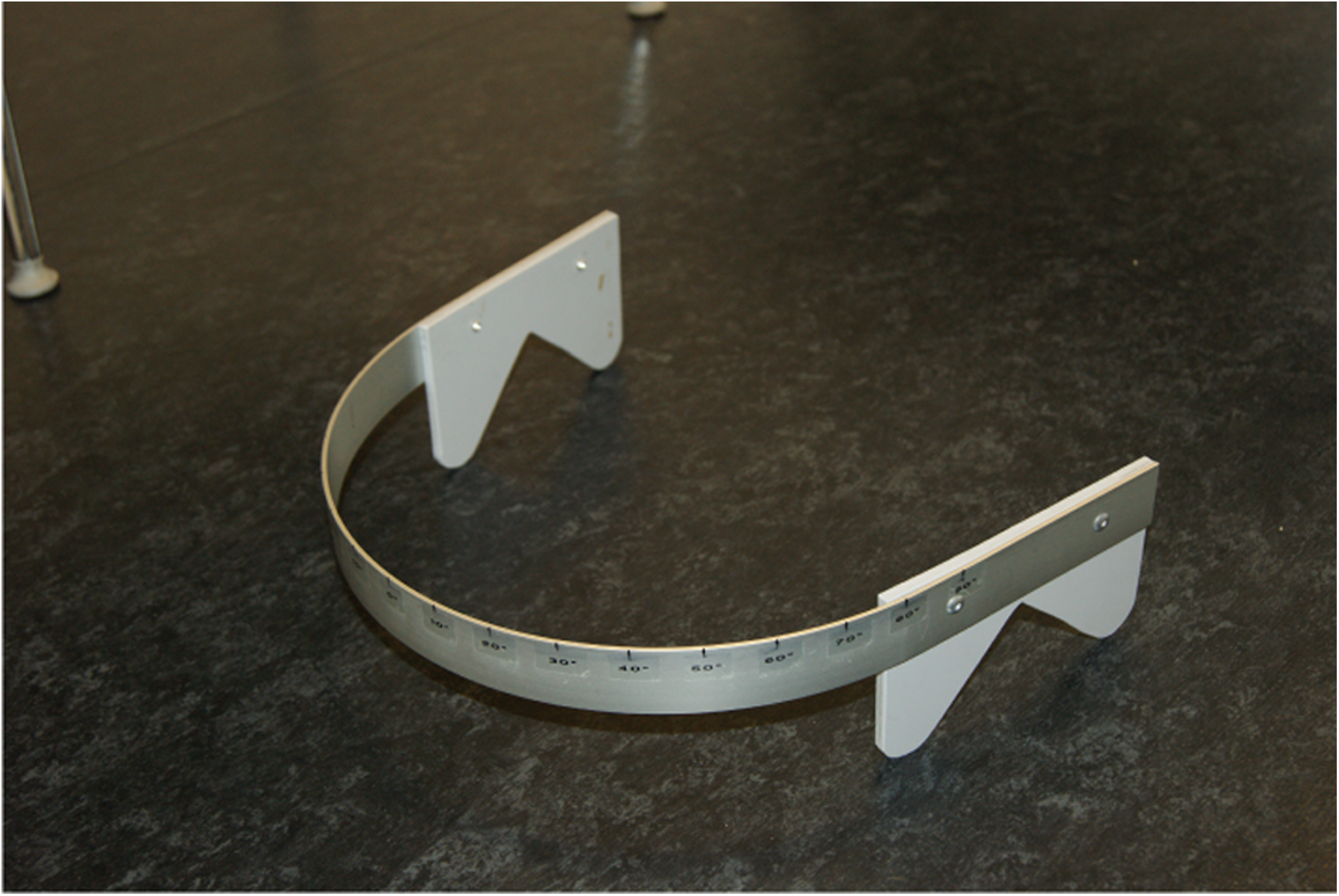

Supplement: Supplementary file 3 — Authors’ original file for figure 2 [file 12891_2014_2400_MOESM3_ESM.tif]

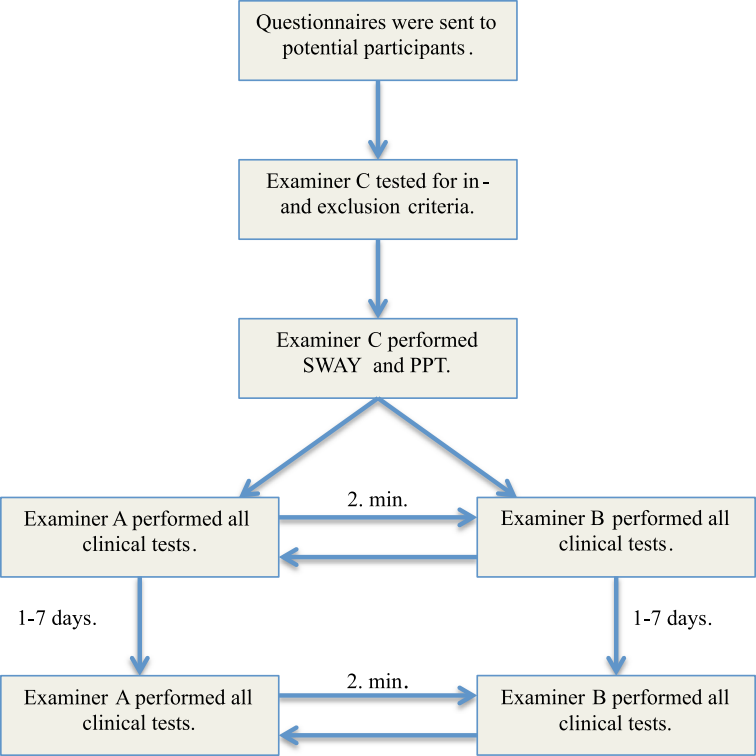

Supplement: Supplementary file 4 — Authors’ original file for figure 3 [file 12891_2014_2400_MOESM4_ESM.pdf]
